# The Prediapause Stage of *Aedes japonicus japonicus* and the Evolution of Embryonic Diapause in Aedini

**DOI:** 10.3390/insects10080222

**Published:** 2019-07-25

**Authors:** Jake Bova, John Soghigian, Sally Paulson

**Affiliations:** 1 Department of Biology, Emory and Henry College, Emory, VA 24327, USA; 2 Department of Entomology and Plant Pathology, North Carolina State University, Raleigh, NC 27607, USA; 3 Department of Entomology, Virginia Tech, Blacksburg, VA 24061, USA

**Keywords:** mosquito, diapause, overwinter, ancestral state reconstruction

## Abstract

The genus *Aedes* is well known for its desiccation-resistant eggs, which frequently serve as an overwintering mechanism through diapause. Despite this, relatively little is known about the diapause and overwintering biology of most *Aedes* species including *Aedes japonicus japonicus*, an invasive mosquito in the United States. The importance of this mosquito in disease systems like La Crosse virus remain uncertain. Embryonic diapause is used by *Ae. j. japonicus* to survive temperate winters, and the persistence of this species in the Appalachian region is a result of overwintering, which has important implications for the transmission of this virus to humans. The objective of this study was to identify the prediapause stage, or the stage sensitive to environmental cues needed to induce diapause in this mosquito. By exposing each *Ae. j. japonicus* life stage independently to short-day photoperiods, we determined that the adult maternal life stage is the prediapause stage. Using the most recent phylogeny and prior literature on the prediapause stages in the genus *Aedes*, we were able to infer the evolutionary history of the prediapause stages of *Aedes* mosquitoes that overwinter or aestivate as eggs. This initial ancestral state reconstruction allowed us to hypothesize that Aedini mosquitoes that undergo obligate diapause may have evolved from those utilizing the embryonic prediapause stage, and that the ancestral prediapause state of Aedini appears to be maternally controlled.

## 1. Introduction

**Figure 1:**
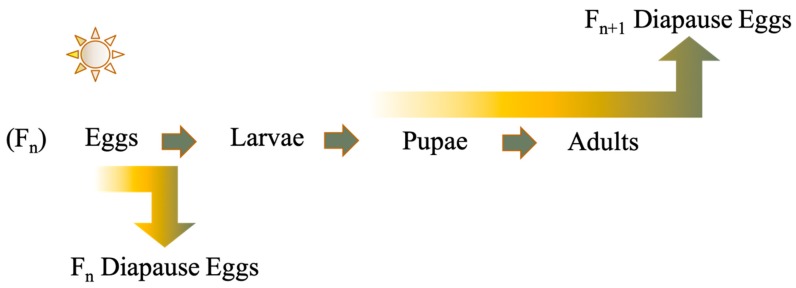
Two common photoperiodic diapause induction methods utilized by facultative embryonic diapause in the genus *Aedes*. Gradient arrows indicate the ability of the life-cycle stage to sense environmental cues needed to enter diapause either in the F_n_ generation or F_n+1_ generation.

Many mosquitoes are faced with adverse weather conditions due to their geographic range, and a major survival strategy is to enter into a hibernal diapause to shield themselves from low temperature extremes. Multivoltine, temperate mosquitoes utilize a facultative diapause usually driven by shorter photoperiods and temperature. Most temperate *Aedes* mosquitoes, as well as those within the tribe they belong to, the Aedini, overwinter as the pharate first instar within their egg, but the life-cycle stage that is sensitive to environmental cues that induce diapause, referred to as the prediapause stage, can differ between *Aedes* species. Either the developing embryo or maternal generation is sensitive to light and temperature to induce embryonic diapause (Figure 1) [1,2,3].

*Aedes japonicus japonicus* is an invasive mosquito in the United States and is capable of vectoring several viruses [4]. This mosquito is a cold-adapted species and has been known to overwinter predominantly as eggs, but some overwintering larvae have been identified [5]. In the Appalachia region, La Crosse virus- and Cache Valley virus-infected pools of *Ae. j. japonicus* have been collected [6,7,8]. Its invasion biology is similar to that of the Asian tiger mosquito *Aedes albopictus.* While a common mosquito in the eastern United States, much remains to be discovered about *Ae. j. japonicus’s* basic biology and its increasingly pertinent vector status makes this imperative to understanding future disease emergence related to this species.

The goal of this study was to identify the prediapause stage of *Ae. j. japonicus* and to better understand the evolution of the diapause system by mapping the known phylogeny for the prediapause stage of *Aedes* mosquitoes that undergo embryonic diapause. Previous discussion on the evolution of diapause has centered around the diapause stage [1]; however, we show here the prediapause phenotype should be considered when evaluating the evolution of embryonic diapause in Aedini.

## 2. Materials and Methods

The *Ae. j. japonicus* we used in this study were collected using modified Reiter gravid traps from a forested area in Montgomery County during the month of July [8]. Adult mosquitoes were allowed to oviposit upon seed germination paper and were then screened for possible viral infection by plaque assay. All mosquitoes were virus-negative.

To elucidate the prediapause stage of *Ae. j. japonicus*, populations of a single life stage of the F_2_ generation were exposed to a 8:16 (L:D) short-day photoperiod, while the remainder F_2_ life stages were exposed to a 16:8 (L:D) long-day photoperiod according to modified methods from Kappus and Venard [9]. As such, the egg, larval, pupal, and adult life stages were all exclusively exposed to the short-day photoperiod, while the additional life stages from the same cohort were exposed to long-day photoperiods, e.g., for mosquitoes treated with short-day photoperiods as larvae, eggs, pupae, and adults of this cohort were exposed to long-day photoperiods. All F_1_ mosquitoes were exposed to long-day photoperiods, including the cohort where eggs were subjected to short-day photoperiods. In addition, groups were raised entirely under short-day photoperiods or entirely under long-day photoperiods as a control, so that all life stages were exposed to either short or long days. All trials were conducted at 21 °C with 75% relative humidity. Adult mosquitoes from the F_2_ generation were force-mated [10], offered a human blood meal via a volunteer inserting their arm in their cages, and the eggs were collected upon seed germination paper (Anchor Paper Company, Saint Paul, MN, USA). After 14 days, 200 eggs randomly selected from the F_3_ cohorts were exposed to a hatching stimulus of 0.5 g of nutrient broth dissolved in 250 mL of deionized water for 24 h. Following the first hatch attempt, the oviposition papers were allowed to dry for another 24 h at a 8 L:16 D short-day photoperiod and were then subjected to a second hatch attempt. Upon completion of the second hatch attempt, we chemically cleared all unhatched eggs using acetic acid and sodium chlorite solution. Eggs were considered embryonated if the pharate first instar larvae eye spots, egg burster, and hatching spine were easily noticeable. Five replicates of F_3_ eggs per F_2_ life stage subjected to diapause conditions were used with the exception of the F_2_ egg treatment. Percent of viable eggs was calculated as: (number of larvae hatched + number of embryonated eggs)/(number of larvae hatched + number of embryonated eggs+ non-embryonated eggs). Percent diapause was calculated as: (no. of embryonated eggs not hatching after two hatching attempts/no. of viable eggs).

*Aedes j. japonicus* prediapause and viability data were analyzed using Chi-square followed by pairwise comparisons of percent hatch for different life stages by Fisher’s exact test using Prism 7 for Mac OSX (GraphPad Software, Inc., 2017, La Jolla, CA, USA).

The evolution of diapause in *Aedes* was assessed by testing for phylogenetic signal in known diapause states and by ancestral state reconstruction of those states across the phylogeny of the Aedini from Soghigian et al. 2017 [11]. This phylogeny was trimmed with the R function drop.tip from the package ape [12] to taxa for which diapause states had been experimentally determined (Table S1). Pagel’s λ [13], a measure of phylogenetic signal, was estimated for these states given the Aedini phylogeny with fitDiscrete in the R package geiger [14]. The resulting model from fitDiscrete was compared to a model assuming no phylogenetic signal (λ = 0) with a likelihood ratio test approximated by a chi-squared distribution in R; a significant difference between these models thus would indicate a better fit of the model with Pagel’s λ and provide evidence of phylogenetic signal in diapause state.

Next, ancestral states were evaluated across nodes of the phylogeny by generating 1000 stochastic character maps with make.simmap from the R package phytools [15] under a model in which transitions between diapause states had an equal rate (model = “ER” in make.simmap), the best fitting model based on corrected Akaike information criterion (AICcs). The posterior probabilities were summarized at nodes to infer the best supported ancestral state for each node and visualized these posterior probabilities as pie charts on the trimmed phylogeny. Where different populations of the same species exhibit different diapause states, an equal probability of that diapause condition was applied for a given species. For *Aedes vexans*, where some populations exhibit facultative diapause but the prediapause condition in terms of maternal or embryonic controls is unknown, equal probabilities for either diapause condition were used in the inference of ancestral states.

## 3. Results

**Figure 2:**
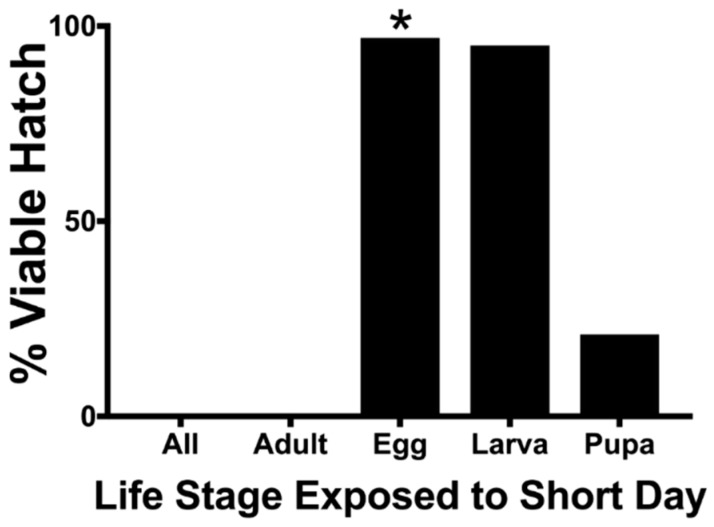
Effect of a short-day photoperiod (8 L:16 D) on *Ae. j. japonicus* F_2_ life stages on F_3_ percent hatch. All other F_2_ life-cycle stages were exposed to a long-day photoperiod (14 L:8 D). * = eggs of the F_2_ generation were tested for percent diapause.

The life stage of a mosquito exposed to short-day photoperiods (8 L:16 D) had a significant effect of the hatch rate of F_3_ eggs (χ^2^, *p* < 0.01) (Table 1 and Figure 2). The exposure of the maternal adult F_2_ generation to short days resulted in F_3_ egg diapause (no hatch) irrespective of all other F_2_ life stage treatments. The hatch rate of eggs produced from egg- and larval-stage exposure to short-day photoperiods was not different from the controls that had not been exposed to any short-day photoperiods (Fisher’s exact test, *p* < 0.01). Short-day treatments on F_2_ pupae elicited a weak diapause response, resulting in a hatch rate that was lower than that of the controls but higher that of the adults that had been exposed to short days (Fisher’s exact test, *p* < 0.01). All larvae maintained at a short-day photoperiod continued development to pupal ecdysis.

Percent egg viability from force-mated pairs of *Ae. j. japonicus* ranged from 50.7 to 58.6% with a total mean of 54.2%, 95% CI (52.9; 55.4) (Table 2) and were not statistically different (Fisher’s exact test *p* > 0.05).

**Figure 3:**
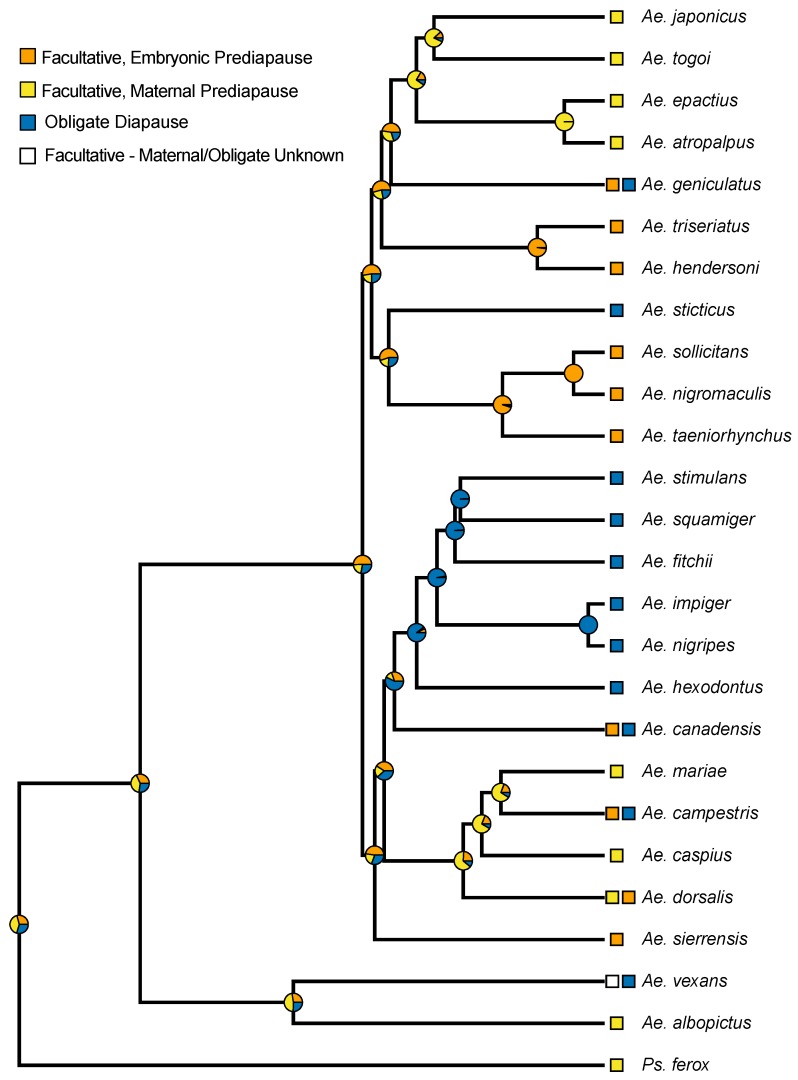
Phylogeny of known prediapause stages in *Aedes* that undergo embryonic diapause. Species with two prediapause stages have obligate univoltine and facultative multivoltine individuals. Some species utilize multiple life-cycle diapause stages and represented here is the prediapause stage solely to induce embryonic diapause. Phylogeny from Soghigian et al. [11], with posterior probabilities from an ancestral state reconstruction shown on nodes.

There was significant evidence of a phylogenetic signal in the prediapause state as the model estimating λ had a significantly better fit than the model with no phylogenetic signal (estimated λ = 1, LR *p* < 0.001). Groupings of similar prediapause phenotypes are noticeable in closely related species; however, they were not noticeable in all species (Figure 3). Maternal prediapause is evident in distantly related species, while embryonic prediapause appears in several clusters of closely related species.

Prediapause Definitions:
Facultative Embryonic: The embryo is sensitive to environmental cues needed to induce diapause and is in the overwintering diapause stage.
Facultative Maternal: The F_n_ maternal female is sensitive to environmental cues needed to induce diapause (Figure 1), while the F_n+1_ embryos enter diapause.
Obligate: These mosquitoes lay eggs that enter into an obligate aestivation to avoid dry seasons, or these mosquitoes lay eggs that enter into an obligate diapause to avoid harsh low temperatures and are located more poleward.
Facultative—Maternal/Obligate Unknown: *Aedes vexans* exhibits an obligate diapause but facultative diapausing populations have been identified; however, the mechanism behind the facultative diapause remains uncertain.

## 4. Discussion

*Aedes j. japonicus* undergoes embryonic diapause facilitated by the prediapause maternal generation that begins detecting environmental cues in the pupal life-cycle stage and extends into the adult life-cycle stage (Table 1 and Figure 2). This maternal prediapause stage is similar in the closely related *Aedes togoi* [16], *Aedes epactius*, and *Aedes atropalpus* [17] and the more distantly related *Aedes albopictus* [18] (Figure 3). Low percentages of viable eggs from force-mated females highlight the challenges of rearing *Ae. j. japonicus* (Table 2). These percentages were consistent across the experiment. With several identifications of virus-infected mosquitoes from the field, a better understanding of the biology of this mosquito is important to understand vector biology and disease transmission [6,7,8].

Recent molecular phylogenetic analyses have made it possible to begin to examine the evolution of the prediapause stages of *Aedes* mosquitoes that undergo hibernal, embryonic diapause. Embryonic diapause also occurs in *Psorophora ferox* and *Anopheles walker* [19,20]. These non-*Aedes* species both employ the maternal prediapause stage to induce embryonic diapause in the following generation. Our ancestral state reconstruction suggests a slightly higher probability relative to other possible states that the ancestral diapause state in the Aedini is maternally controlled, diapausing eggs (See Supplemental Figure S1). Due to how little is known about the prediapause condition in the majority of Aedes species, our ancestral state reconstruction, and the conclusions from it, should be considered preliminary until further research on diapause states in additional species can be performed and evaluated within an evolutionary context. That said, beyond the higher posterior probability at basal nodes for a maternal prediapause stage, an ancestral state of facultative maternal prediapuse in the Aedini is consistent with the fact the basal lineage of the Aedini is *Psorophora*, and many *Psorophora* species have been described as multivoltine and overwintering in the egg [21]. Multivoltine species with such an overwintering strategy must have facultative diapause, but *Psorophora ferox* is the only species to our knowledge within *Psorophora* where this facultative diapause has been further tested to reveal a maternal pre-diapause condition. Thus, our results are consistent with an ancestral state for the Aedini that involves a prediapause stage that is maternally controlled [2,11].

Our results suggest that the evolution of an obligate diapause condition may be preceded by the evolution of facultative embryonic prediapause. All species exhibiting an obligate diapause state, other than *Aedes vexans*, have an ancestral node with a high probability of embryonic prediapause, or themselves may exhibit embryonic prediapause in some populations. As with considerations for the ancestral condition of prediapause in the Aedini, this observation is highly preliminary and warrants further consideration as additional studies are conducted on diapause.

In *Aedes campestris* [22], *Aedes geniculatis* [23], and *Aedes canadensis* [20], all species have individuals that undergo obligate embryonic diapause to survive northern winters and are univoltine, but more southern populations have individuals capable of facultative embryonic diapause and are multivoltine. While more distantly related, this strategy appears to be convergent, and may indicate that diapause behaviors could be variable across populations of other species, as well. This variability could be a strategy triggered by a warming climate. The studies on the facultative diapause populations of these species date back several decades and merit more current scrutiny along with other poleward species.

## 5. Conclusions

With the addition of identifying the maternal prediapause stage of *Ae. j. japonicus* and subsequent phylogenetic analysis, we add basic knowledge to the US invasive *Ae. j. japonicus*, and we present a more detailed understanding of the evolution of embryonic diapause in *Aedes* mosquitoes. Our preliminary analysis indicates that embryonic prediapause precedes obligate diapause and the ancestral diapause state of Aedini is maternally controlled. Further and more recent sampling of diapause and prediapause states are needed to better understand this fascinating system.

## Figures and Tables

**Table 1:** Effect of a short-day photoperiod (8 L:16 D) on *Aedes japonicus japonicus* F_2_ life stages on F_3_ percent diapause. All other F_2_ life-cycle stages were exposed to a long-day photoperiod (14 L:8 D). * = eggs of the F_2_ generation were tested for percent diapause. Percentages followed by different letters are significantly different (Fisher’s exact test, *p* ≤ 0.01).

Life-Cycle Stage	*n* Eggs	% Diapause
All	521	99.81 A
Adults	532	100 A
Eggs	507 *	2.96 B
Larvae	536	4.66 B
Pupae	586	20.82 C
None	568	2.82 B

**Table 2:** Percent viability of force-mated *Ae. j. japonicus* eggs by life stage exposed to a short-day photoperiod (8 L:16 D). Viability was determined as bleached eggs that display noticeable eye spots and egg bursters. *Ae. j. japonicus* males and females were force mated according to Gerberg et al. 1994 [10].

Life Stage Exposed to 8 L:16 D	% Viability of Eggs
ALL	52.1
Adult	53.2
Pupa	58.6
Larva	53.6
Egg	50.7
None	56.8

## Supplementary Materials

The following are available online at https://www.mdpi.com/2075-4450/10/8/222/s1, Table S1: Description of species, prediapause stage to induce embryonic diapause, and references; Figure S1: Posterior probabilities of nodes for Figure 3.
