# Supplementary material for: The Prediapause Stage of Aedes japonicus japonicus and the Evolution of Embryonic Diapause in Aedini
Source: Insects. 2019 Jul 25;10(8):222. doi: 10.3390/insects10080222 (PMC6723955; doi:10.3390/insects10080222)
Supplement: Supplementary file 1 [file insects-10-00222-s001.zip › Supplementary/Table S1.docx]

**Table S1.** Description of species, prediapause stage to induce embryonic diapause, and references

***Aedes albopictus* Maternal**

Mori, A.; Oda, T.; Wada, Y. Studies on the egg diapause and overwintering of *Aedes albopictus* in Nagasaki. *Trop. Med*. **1981**, *23,* 79‐90.

***Aedes atropalpus* Maternal**

Anderson, J.F. Influence of photoperiod and temperature on the induction of diapause in *Aedes atropalpus* (Diptera, Culicidae). *Entomol. Exp. Appl.* **1968,** *11*, 321‐30

***Aedes campestris* Embryonic**

Tauthong, P.; Brust, R.A. The effect of photoperiod on diapause induction, and temperature on diapause termination in embryos of *Aedes campestris* Dyar and Knab (Diptera: Culicidae). *Can. J. Zool*. **1977**, *55,* 129‐34.

***Aedes canadensis* Embryonic**

Pinger, R.R.; Eldrige, B.F. The effect of photoperiod on diapause induction in *Aedes canadensis* and *Psorophora ferox* (Diptera: Culicidae). *Ann. Entomol. Soc. Amer*. **1977**, *70*, 437‐ 41.

***Aedes caspius*  Maternal**

Abdel‐Rahman, A.M.; Adham, F.K. The effect of photoperiod on diapause induction in *Aedes caspius* Pallus. *J. Egyptian Soc. Parasitol*. **1983,***13*, 343‐47.

Vinogradova, E.B. Role of photoperiodism and temperature in the induction of diapause in the egg phase of *Aedes caspius caspius* Pallus. (Diptera, Culicidae). *Parazitologiia* **1975**, *9(*5), 385-392.

***Aedes dorsalis* Embryonic, Maternal = temperature dependent**

Telford, A.D. The pasture *Aedes* of central and northern California. Seasonal history. *Ann. Entomol. Soc. Amer*. 1958, *51*, 360‐65.

McHaffey, D. G.; Harwood, R. F. Photoperiod and temperature influence on diapause in eggs of floodwater mosquito, *Aedes dorsalis* (Meigen) (Diptera: Culicidae). *J Med Entomol*. **1970**, *7*, 631-44.

***Aedes epactius* Maternal**

Anderson J.F. Influence of photoperiod and temperature on the induction of diapause in *Aedes atropalpus* (Diptera, Culicidae). *Entomol. Exp. Appl*. **1968**, *11*, 321 – 30.

***Aedes fitchii* Obligate, Flood**

Horsfall, W.R.; Lum, P.T.M.; Henderson, L.M. Eggs of floodwater mosquito (Diptera: Culicidae) V. Effect of oxygen on hatching of intact eggs. *Ann. Entomol. Soc. Amer.* **1958**, *51*, 209‐13.

***Aedes geniculatus* Embryonic and Obligate, Winter**

Sims, S.R.M.; Munstermann, L.E. Egg and larval diapause in two populations of *Aedes geniculatus* (Diptera: Culicidae). *J Med Entomol*. **1983** *3*, 263‐71.

***Aedes hendersonii* Embryonic**

Gallaway, W.J. Larval diapause of *Aedes hendersoni* and *Aedes trisereatus* from southern Manitoba. *J. Am Mosq Control Assoc.* **1985**, *1*, 92‐93.

Shroyer, D. A. Seasonal aspects of egg hatching in *Aedes triseriatus* (Say): sex ratio distortion and diapause. Ph.D. diss. Univ. of Notre Dame, Notre Dame, IN. **1979**. 181 pp.

***Aedes hexodontus* Obligate, Winter**

Beckel, W.E. Investigations of permeability, diapause, and hatching in eggs of the mosquito *Aedes hexodontus* Dyar. *Can J Zool*. **1958**, *36*, 541‐55.

***Aedes impiger*  Obligate, Winter**

Corbet, P.S.; Danks, H.V. Egg‐laying habits of mosquitoes in the high arctic. *Mosq. News.* **1975**, *35*,8‐14.

Corbet, P.S.; Danks, H.V. Seasonal emergence and activity of mosquitoes (Diptera:Culicidae) in a high‐artic locality. *Can Entomol*. **1973**, *105*, 837‐72.

***Aedes japonicus japonicus* Maternal**

This paper

***Aedes mariae*  Maternal**

Coluzzi, M.; Di Deco, M.; Gironi, A. The influence of photoperiod on the selection of oviposition sites in *Aedes mariae* (Diptera: Culicidae). Parassitologia. **1975**, *17*, 121‐30.

***Aedes nigripes* Obligate, Winter**

Corbet, P.S.; Danks, H.V. Egg‐laying habits of mosquitoes in the high arctic. *Mosq. News.* **1975**, *35*,8‐14

Corbet, P.S.; Danks, H.V. Seasonal emergence and activity of mosquitoes (Diptera:Culicidae) in a high‐artic locality. *Can Entomol.* **1973**, *105*, 837‐72.

***Aedes nigromaculis* Embryonic, temperature dependent**

Telford, A.D. The pasture *Aedes* of central and northern California. Seasonal history. *Ann. Entomol. Soc. Amer*. **1958,** *51,* 360‐65.

***Aedes sierrensis* Embryonic, primarily L4 larvae**

Jordan, R.G.; Bradshaw, W.E. Geographic variation in the photoperiodic response of the western tree‐hole mosquito, *Aedes sierrensis*. *Ann. Entomol. Soc. Amer*. **1978**, *71,* 787‐90.

Jordan, R.G. Embryonic diapause in three populations of the western tree‐hole mosquito, *Aedes sierrensis*. *Ann. Entomol. Soc. Amer*. **1980,** *73,* 357‐59.

***Aedes sollicitans* Embryonic**

Parker, B.M. Photoperiod‐induced diapause in a North Carolina strain of *Aedes sollicitans*; photosensitivity of fully formed and developing embryos. *J. Am Mosq Control Assoc.* **1988**, 4, 57‐63.

***Aedes squamiger* Obligate, Flood**

Telford, A.D. The pasture *Aedes* of central and northern California. Seasonal history. *Ann. Entomol. Soc. Amer*. **1958**, *51*, 360‐65.

***Aedes sticticus* Obligate, Flood**

Horsfall, W.R.; Trpis, M. Eggs of floodwater mosquitoes. X. Conditioning and hatching of winterized eggs of *Aedes sticticus* (Diptera: Culicidae). *Ann. Entomol. Soc. Amer*. **1967,** *60*,1021‐25.

***Aedes stimulans* Obligate, Flood**

Horsfall, W.R.; Fowler, H.W.J. Eggs of floodwater mosquitoes VIII. Effect of serial temperatures on conditioning of eggs of *Aedes stimulans* Walker (Diptera: Culicidae). *Ann. Entomol. Soc Amer*. **1961,** *54*, 664‐66.

***Aedes taeniorhynchus* Embryonic**

Parker, B.M. Effects of photoperiod on the induction of embryonic diapause in *Aedes taeniorhynchus* (Diptera:Culicidae). *J Med Entomol*. **1985**, *22*, 392‐97.

***Aedes togoi* Maternal**

Galka, B. E.; Brust, R. A. The effect of temperature and photoperiod on the induction of larval diapause in the mosquito *Aedes togoi* (Theobald) (Diptera: Culicidae). *Can J Zool*. **1987**, *65*(9), 2262-2265.

***Aedes triseriatus* Embryonic**

Kappus, K.D.; Venard, C.E. The effects of photoperiod and temperature on the induction of diapause in *Aedes triseriatus* Say. *J. Insect Physiol*. **1967**, *13*, 1007‐19.

***Aedes vexans* Obligate, Flood**

Wilson, G.R.; Horsfall, W.R. Eggs of floodwater mosquitoes XII. Installment hatching of *Aedes vexans* (Diptera: Culicidae). *Ann. Entomol. Soc. Amer*. **1970**, *63*, 1644‐47.

***Psorophora ferox* Maternal**

Pinger, R.R.; Eldridge, B.F. The effect of photoperiod on diapause induction in *Aedes canadensis* and *Psorophora ferox* (Diptera:Culicidae). *Ann. Entomol. Soc. Amer.* **1977**, *70*, 437–441.
