# Supplementary figures and images for: The Prediapause Stage of Aedes japonicus japonicus and the Evolution of Embryonic Diapause in Aedini
Source: Insects. 2019 Jul 25;10(8):222. doi: 10.3390/insects10080222 (PMC6723955; doi:10.3390/insects10080222)

Posterior Probabilities for States at Each Node

| Node Num | Embryonic | Maternal | Obligate |
|----------|-----------|----------|----------|
| 27       | 0.288     | 0.419    | 0.293    |
| 28       | 0.317     | 0.412    | 0.271    |
| 29       | 0.272     | 0.475    | 0.253    |
| 30       | 0.504     | 0.217    | 0.279    |
| 31       | 0.478     | 0.215    | 0.307    |
| 32       | 0.406     | 0.21     | 0.384    |
| 33       | 0.243     | 0.648    | 0.109    |
| 34       | 0.189     | 0.729    | 0.082    |
| 35       | 0.193     | 0.717    | 0.09     |
| 36       | 0.303     | 0.124    | 0.573    |
| 37       | 0.074     | 0.034    | 0.892    |
| 38       | 0.023     | 0.005    | 0.972    |
| 39       | 0         | 0        | 1        |
| 40       | 0.008     | 0.002    | 0.99     |
| 41       | 0.006     | 0.002    | 0.992    |
| 42       | 0.53      | 0.22     | 0.25     |
| 43       | 0.551     | 0.191    | 0.258    |
| 44       | 0.95      | 0.022    | 0.028    |
| 45       | 1         | 0        | 0        |
| 46       | 0.541     | 0.239    | 0.22     |
| 47       | 0.979     | 0.011    | 0.01     |
| 48       | 0.475     | 0.321    | 0.204    |
| 49       | 0.166     | 0.738    | 0.096    |
| 50       | 0.002     | 0.995    | 0.003    |
| 51       | 0.117     | 0.815    | 0.068    |

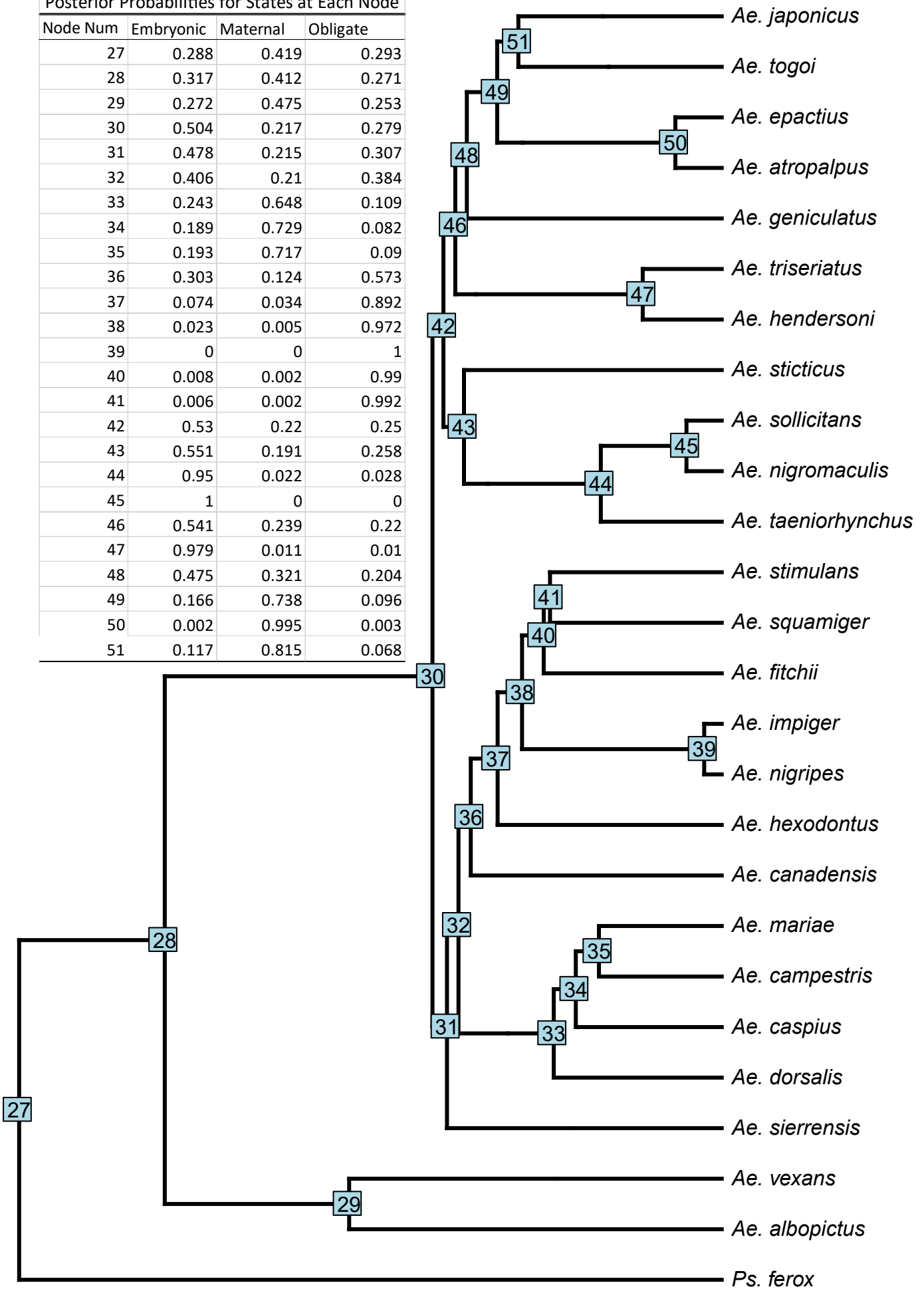

Supplement: Supplementary file 1 [file insects-10-00222-s001.zip › Supplementary/Figure S1.pdf]
